# Leçons De Physiologie Expérimentale, Appliquée a La Médecine, Faites Au Collége De France

**Published:** 1857-07

**Authors:** 


ART. III.—*Leçons de Physiologie Expérimentale, Appliquée a la Médecine, faites au Collége de France.* Par M. CLAUDE BERNARD, Membre de l'Institut de France. Prof. de Médecine au Collége de France. Prof. de Physiologie à la Faculté des Sciences, &c. &c. Tome Deuxième. Cours du Semestre d'été, 1855. Avec 78 figures intercalées dans le texte.

No faculty necessary to make a great physiologist seems to have been denied to the author of the work before us. For ten years we have been accustomed to hear the name of Bernard coupled with discoveries of the utmost value to medicine, and have learned to listen with respect and interest to all that comes from his lips. Certainly, a more remarkable union of the inductive and experimental powers has rarely been met with in the same mind, and still more rarely have they been made use of with an industry so untiring, and a success so uniform.

Were any other proof wanting of the possession, by Claude Bernard, of the rarest intellectual gifts, the volume, whose title heads our page, would of itself be an ample warrant of the truth of our assertion.

In a brief introduction, the author points out the two modes of teaching which are at present in vogue. The one which is usually adopted in our colleges, consists in a dogmatic statement of the positive and applicable facts of a science. While the professor strings these together, bead-like, upon the thread of some accepted theory, he troubles the young student but little with the many doubts and difficulties which render medical opinions so changing, and often so short-lived. No doubt such a method of instruction makes the task of the beginner easy, but, on the other hand, it possesses this disadvantage, that he is liable to find himself, at the close of his curriculum, so fixed in certain views, that he is fortunate if the yearly changes in science do not find him indisposed to bend to the legitimate alterations which they are constantly effecting in the modes of thought and action of wiser, or more flexible minds. The other mode of teaching is, without doubt, better adapted to those who either find pleasure in a frank statement of all the facts of a given case, or who, themselves, are to be actively engaged in original research or study. That the method chosen by M. Bernard is well adapted for these latter purposes, we hope to show as we proceed, while, at the same time, it will be seen to have certain defects, inseparable, perhaps, from the very form of instruction.

In the volume before us, our author has given us, in the form of lectures, the results of a series of researches upon the glands, whose secretions take part in the digestive processes. No mere statement of his conclusions can give any idea of the charm inseparable from his manner of imparting his ideas. Thus, in place of a bare narration of his views, together with the facts, old or new, upon which they rest, he invites the reader, as it were, to enter his workshop, and view with him the action of the machinery by which these results are attained. For example, some point of physiological interest arises. It is pursued through its relations to chemistry, mechanics, and anatomy. Experiments upon animals, ingeniously planned, create new facts. The doubts, the difficulties, the failures are made present to the reader, or the hearer. The analyses are shown to the class, and finally, the resultant post-mortems are exhibited. Nay, the very disappointments which so often wait upon experimental inquiries, become the starting-points of new researches, and every link in the connected chain of reasoning is brought palpably before us. So aided, we sometimes accompany the teacher, in a single lecture, over researches which in some cases have extended through many laborious years. The pleasure and privilege of being thus admitted to view the mechanism of such a mind as that of M. Bernard, is not the least charm of his book. Appearing to recognize instinctively the delight with which we seek to learn how discoveries are made, he invites us to the closest psychological intimacy, so that we see, as we follow him, how he came to think of this or that point, and why he made such and such an experiment, and how it failed, and why it failed, and how then he varied the conditions, and began anew. Such a method of relation and teaching may be unfitted for the ordinary learner, but for the physician, it gives to the story of a scientific research, the variety and fascination of a voyage of adventure.

In the first lecture M. Bernard discusses some of the fallacies which seem to belong to the ordinary methods of physiological investigation, and points out that which he believes to be the best means of discovering the secrets of the human frame. Among the fallacies thus alluded to, is that of attempting to discover the function of an organ by mere inspection of its anatomical arrangements. The acceptance of such evidence singly, has been fertile of error. It is rare that mere structural study of dead tissues, however aided by the lens, will enable us to assign to any given organ a certain function. Anatomy can but analyze the machinery when the works have stopped; and what more than a fair guess could we make as to the uses of a complex machine, which we saw only in a state of rest? Give to it the life of motion, and it will often explain itself, at once, to the eye of educated intelligence. How far more complicated than any mechanism of mere human design, is that of the simplest animal function, we need hardly remind the reader. If then we are to study successfully the workshops and factories of the body, we must study them while active and alive, and laying aside that mode of investigation which contents itself with questioning form as to function, must resort to quite a different mode of research. Such a method, which M. Bernard terms the physiological, or functional, as distinguished from the mere anatomical, consists in the pursuit of a function or vital action throughout the organism, until, by sufficient evidence, we are able to localize it in some particular organ, or set of organs. Thus we end by fitting the vital act to the organ, whereas, by the other method, we fit the organ to the act.

"You have no doubt, observed," says our author, "that in our researches, we have not been guided by any preconceived ideas of anatomical localization, and that it was not by asking what might be the functions of the liver, that we have arrived at this discovery." (That of the glycogenic function of the liver.) On the contrary, in our mode of procedure, the anatomical localization of a function has been but a result of physiological investigations. We have followed a phenomenon, that of the diverse mutations of sugar in the animal, and have thus arrived at the knowledge of a fact, hitherto unknown, namely, that the liver of man and animals is constantly engaged in the formation of this substance. To this mode of investigation, anatomical knowledge is only incidentally necessary. As it deals with life, it demands the aid of vivisection at every step. The knife of the operator lays bare the living, active organ, and enables us, as M. Bernard has elsewhere remarked, to carry the torch of analytic chemistry into the midst of an organism still throbbing with vitality. An exposition by lectures of a research so conducted, brings us, as it were, face to face with phenomena, and might almost be entitled to the name of clinical physiology.

Chapter Second treats of thehistory of the anatomy of the glands, whose secretions enter the mouth, of their comparative anatomy, and, in fact, of all that can be learned by mere inspection, however skilled. We are here reminded, again, how negative is the evidence of anatomy as to the functional distinctions between the several salivary glands. A parotid cell resembles indifferently a cell from the submaxillary or sublingual glands, and is equally like those which make up the pancreatic tissue. On the other hand, physiology readily succeeds in assigning to these several glands offices which no anatomical skill could suspect them to possess.

With this assurance, M. Bernard begins with us the study of the parotid secretion, as the most important of those furnished by the salivary glands. In looking for a clue to conduct us into this research, we are struck with the fact that the parotid gland accompanies the possession of teeth, and that, in any given animal, its size bears a certain relation to the importance of mastication. Thus, the birds who do not crush their food, have no parotid glands. The ruminants have it amply developed. The carnivora, who are hasty feeders, and "bolt" their food, have but small parotids, while marine animals, as the seal, whose food is always watery enough, have this gland far less developed than we find it in the terrene animals who most nearly approach them in structure.

The hint thus given leads us to suspect the existence of some relation between the amount of water in food, the extent of mastication which it exacts, and the supply furnished by the gland under study. This suspicion is answered affirmatively by the results of experiments, some of them old, and some recent. We learn thus, that the drier the food, and the more chewing it requires, the larger is the supply of parotid fluid: that it flows freely during mastication, and ceases to flow afterwards, and that, if we chew on the left side, the left parotid is the most active, and that when we chew upon the right side the reverse occurs. Finally, the fish, since he lives in water, has hardly any parotid saliva, and in land animals, when drinking, the gland ceases to act.

It was long believed that these various effects were due to the pressures, etc., exerted during mastication by the muscles engaged in the process. The insufficiency of any such idea to explain the facts above stated, as well as the influence of emotions upon the salivary flow, induced M. Bernard to test the governing powers of the nerves over this secretion. Galvanization of the inferior maxillary branch of the fifth pair of nerves, or the facial portion of the seventh, was found to augment the flow of parotid saliva, upon the side whose nerves were thus excited. Combining this evidence, it seems to be clear that the fluid of this gland is intended to render mastication more easy, being made use of mechanically, to moisten and soften the food during this action, so as to facilitate the digestive processes which follow. Digestion, indeed, can be shown to be more or less productive of nutritive results according as the food is more or less completely comminuted.

In physical and chemical composition, as has long been known, the saliva of the parotid is merely an aqueous fluid, always alkaline, and containing some little saline and albuminous matters.

The next lecture begins with a description of the mode of producing a fistula of the submaxillary gland, an experiment which is original with M. Bernard. The operative procedure is described with extreme minuteness, and here, as elsewhere, the age and appearance of the physiological patient are dwelt upon, and even his conduct, under these rather trying circumstances, described with care and patience. We are especially struck with the fact, more than once alluded to, that "the animal did not seem to suffer." Whether the dog Parisian is deficient in powers of expressing his feelings, or whether a very commendable respect for science, and for M. Bernard, restrains his emotions, we confess ourselves unable to say.

The fluid obtained through the artificial fistula of the submaxillary gland is totally different from that of the parotid. As it flows drop by drop from a tube placed in the duct of Wharton, we observe its viscid nature and its highly alkaline reaction, while, on cooling, we see no such deposit of carbonate of lime as usually occurs in parotid saliva. When heated, it swells, but does not coagulate. What, then, is the use of this viscous alkaline fluid? A hint as to its office is obtained by observing how it leaps in jets from the duct of Wharton, when we are strongly impressed with the smell, or even the thought of some agreeable sapid body. Has this gland, then, any connection with the sense of taste? To answer this question, another unlucky dog is chosen. Tubes are slipped into his parotid and submaxillary ducts, and the lingual branch of the fifth pair of nerves is exposed to view. This nerve-twig, commonly regarded as the nerve of taste, is now divided. Galvanic irritation of the peripheral portion gives no result. Like irritation of the central end, produces at once a flow of saliva from the submaxillary gland, but none at all from the parotid. This lingual nerve being the nerve of gustation, any irritant acting on the portion still in connection with the brain, will despatch to that organ an impression of such a character as would ordinarily have reached it when the papilla of the tongue were stimulated by a sapid substance. The reflex impression, which sets out from the brain, and finally stimulates the gland to act, is conveyed through the chorda tympani nerve to the submaxillary ganglion. In proof of this, direct irritation of this ganglion likewise stimulates the gland. When the tissue of the gland is directly galvanized, no effect is induced; so that the intermediation of nerves appears to be essential.

To complete this chain of evidence, M. Bernard proceeds to show that certain gustatory impressions exert a very curious influence upon the flow of the various salivary juices. For this purpose, another canine victim is provided, and artificial exits created for each of the principal salivary fluids, —parotid, submaxillary, and sublingual. Vinegar, aloes, quinine, &c., are then placed successively upon the tongue. It is found that in each case, the submaxillary saliva flows freely, while but little runs from the other glands. Water does not seem to increase the flow from any of the glands. Finally, the proof of connection between the sense of gustation and the tide of submaxillary secretion, is strengthened by the fact that the animals who chew sapid food—the carnivora, for example—possess large submaxillary glands; while in the grain-eating birds, whose food is comparatively insipid, this gland almost disappears from anatomical view.

The sublingual gland is studied in the same manner as the larger glands, and, together with the gland of Nuck, is grouped by M. Bernard along with the mucous or muciparous glands of the mouth. Like them, these two glands secrete a glutinous fluid, which is unlike the thin parotid juice, and which is also separable from the submaxillary fluid, by the fact that it gelatinizes upon cooling.

In connection with the salivary glands, we notice some extremely ingenious experiments upon the elective elimination of certain substances from the blood.

If, for instance, yellow prussiate of potash, iodide of potassium, and grape sugar be injected at the same time into the blood, the iodide appears in the saliva at once; the prussiate in the urine alone, in seven minutes; and the sugar in the same fluid, in twenty-five; at the end of three hours, the iodide can be found in the urine; but neither the sugar, nor the prussiate, are at any after-time appreciable in the saliva. The lactate of iron is another salt which never passes from the blood into the saliva. Iodide of iron, however, is readily eliminated by this route; when therefore, a solution of lactate of iron is placed in the stomach, it is soon discoverable in the blood, but does not pass out with the saliva; if iodide of potassium be now thrown into the same stomach, decomposition occurs, iodide of iron is formed, and within a few minutes is discoverable by tests in the saliva. If, in place of being thus brought into contact in the stomach, these two salts meet in the blood; no decomposition occurs, and the iodide alone is eliminated by the salivary glands. This influence of the blood in preventing certain chemical combinations which take place with facility in the stomach, is a fact which has other examples than the one now stated, and is well deserving of more ample attention than it has hitherto received.* The remaining experiments in the same connection, prove that the salivary ducts are capable of absorbing certain substances with great ease; owing to this, the right parotid gland may be absorbing iodide of potassium injected into its ducts, while the other parotid is eliminating the same salt from the blood. Still more curious is the fact that prussiate of potash is easily taken in by this route, but, as we have seen, is never excreted by it. The only exception to these laws of elective elimination occurs when the blood is so loaded with *any* soluble salt as to have its composition considerably altered, and to subject to serious accidents, the being in whose vessels it circulates. Under these circumstances, even prussiate of potash may appear in the saliva, as well as in the urine.

We cannot avoid agreeing with M. Bernard in his remarks at the close of this chapter, upon the fallacies which have hitherto surrounded every attempt to calculate the whole daily amount of the salivary juices. In fact, we have not as yet the elements for such a calculation; nor is it safe to apply to one animal as man, the results obtained from experiments upon another, as the dog. If indeed, the flow of saliva varies with the activity of mastication and digestion, with every new gustatory impression, and even with the period of the day, while at the same time the flow from a right parotid gland is no index of what the left one is doing, how vain must be the attempt to calculate the flow of a day's saliva during one hour, and then multiplying this result by the hours of the day, and observing how much saliva is formed for each pound of weight, to fit the result to man, by a comparison of his weight with that of the dog thus treated. The same kind of criticism applies equally well to all the estimates of this kind hitherto made. The calculation of Bidder and Schmidt states the amount of saliva at about 20 oz daily, for a man of ordinary weight. If this be anywhere near the real quantity under common circumstances, what amount a tall Western student must evolve under the joint effect of six hours of lectures, and unlimited tobacco, is a question of considerable difficulty. The conversion of the pine floors of our lecture rooms into fine dark mahogany, would, we think, speak volumes, not figuratively, but literally, as to the maximum possibilities in the way of salivary supply, upon this side of the Atlantic at least. Professor Draper, of New York, who seems to have a Briton's aversion to our national habit, in speaking of its effect upon the chemical constitution of saliva, makes some pleasant

* Notes on M. Bernard's lectures on the blood; with an appendix. By Walter F. Atlee, M.D., Philadelphia, 1854, p. 32.

little suggestions to the brethren of the weed, which we recommend to their grateful attention.*

Chapter Sixth completes the subject of the salivary functions. Being in the form of a lecture, it discusses certain minor and disconnected facts, as they suggest themselves to the speaker. The most curious of these wayside discussions concerns the long-mooted existence of sulphocyanide of potassium in the saliva. Those who desire to see the previous history of the subject, will find it in Lehman's Animal Chemistry, and more especially in the additions at the end of the third volume, London, 1854. M. Bernard's researches are of a still later date, and, as they contradict more or less a large number of German researches, are likely to be pretty freely canvassed. On the whole, M. Bernard agrees with Berzelius, in denying the existence of cyanogen in perfectly healthy saliva. Nevertheless, he thinks it probable, that many accidental circumstances, such as carious teeth, may determine its production, and on the whole, leaves the subject in very considerable doubt. We observe that he still speaks of the sulphocyanide as a very poisonous body. We supposed that the contrary had been long ago established by Marchand, and still more lately, by the direct experiments of Wohler and Frerichs. At a time when it was generally considered to be an active poison, its presence in the saliva was the source of many very wild speculations in connection with hydrophobia.† Eberle believed, that saliva, passed under the influence of agreeable thoughts, was free from sulphocyanide, while that which flows when we ponder over unpleasant things, or think about our enemies, becomes instantly charged with the salt in question.

The views of M. Bernard in regard to the formation of tartar on the teeth are altogether original, and we think hardly tenable. In his opinion, the tartar is caused by "an irritation of the alveolo-dental periosteum, owing to the pushing down of the protecting gums by fragments of food during mastication." Now, tartar contains from 60 to 80 per cent. of phosphate of lime, with 10 or 20 per cent. of animal matters; while mixed saliva contains so little of the lime salt, that in some analyses it is set down as "a trace," or neglected altogether. On this account, M. Bernard considers as insufficient, the ordinary explanation which ascribes the formation of tartar to an evaporation, or decomposition of the mixed saliva, and advances the theory above stated. He therefore accounts for the formation of tartar, by regarding it as an abnormal secretion from the denuded dental periosteum. We fancy that our dentists will scarcely coincide in this opinion.

It is within every one's experience, that tartar forms not alone at the alveolo-dental margin, but higher up, and also upon the lateral angles of

* Draper's Physiology, p. 47.

† Wright, in the Lancet, 1842.

the teeth, while the deposit occurs, not from below, but on top of the tartar already deposited, so as sometimes to constitute considerable masses of calcareous matter. How then can it have the origin which M. Bernard assigns to it? Every one is aware, too, that it is formed most largely at night, when, as gustation and mastication are in abeyance, the saliva flows in minimum amount. This, together with the other considerations advanced by M. Bernard, enables us to follow him so far as to admit that the deposit of tartar from the saliva *alone*, is at the least improbable. During the night, however, the mouth is chiefly lubricated by the buccal mucus. That it contains phosphate of lime, the analyses of Bidder and Schmidt sufficiently prove. Is this not a possible source of tartar, or, at least, an additional source? That it, or the saliva, contains but a small amount, hardly weakens the case. Oxalate of lime is found in urine in quantities so minute, as to baffle quantitative determination. Yet calculi of oxalate of lime are only too common. When we remember at the same time, that tartar is freely deposited upon artificial teeth, and when we add to this, that every cause productive of pathological changes in the buccal mucous membrane, increases the deposit of tartar, and when we are reminded at the same time how the phosphate of lime element increases in diseased mucous discharges generally, we shall see that M. Bernard's view of this matter is at least not so perfect as to be above all critical attack.

In concluding his admirable study of the saliva, M. Bernard especially insists on its mechanical relations to mastication, gustation, and deglutition. In the thin parotid fluid he recognizes a liquid of the utmost utility, as an aid to mastication, while in the other and thicker juices of the various glands, he sees antifrictive and lubricating agents calculated to facilitate the action of swallowing.

The vexed question of the conversion of starch into grape sugar, concludes this lecture. M. Bernard is of opinion that "the role of saliva in the chemistry of digestion is insignificant, if not altogether null." It is certainly true that the buccal mucus, and even the pure saliva of the parotid gland will convert *boiled* starch into grape sugar; but *boiled* starch is not the common food of animals, and even if it were, since the gastric acid effectually checks this change, the time for any such conversion would be but brief; being limited to the period occupied in mastication and deglutition. Consequently, our author infers that the mixed saliva has but little chemical utility in digestion, and that, as this necessary conversion of starch into grape sugar is provided for elsewhere, not only as regards boiled, but also raw starch, we are justified in regarding the saliva as fulfilling no other functions but those of a purely mechanical nature.

M. Bernard next proceeds to the study of the pancreas, the so-called abdominal salivary gland. Its anatomical and physiological history receive a brief attention, and we are thus brought up to the date of 1846, in which year our author first directed his mind to the character and uses of its secretion. In accordance with the initial precepts of this volume, the anatomy of the pancreas is first studied, not as a primary means of discovering its function, but as a proper and cautious preparation for the numerous vivisections which are to guide us to the truth. The chief useful anatomical fact which we thus acquire, is that man, and many animals possess at least two pancreatic ducts; and that in birds this organ is multiple. We are thus prepared to follow the lecturer in his experimental illustrations of what he believes to be the function of the pancreas.

Long previous to 1846, Doctor, now Sir Richard Bright, of London, detailed a series of cases, by means of which he established a relation between the existence of fatty diarrhœa, and that of pancreatic disease. Dr. Bright did not avail himself further of the hint thus afforded, and so failed to add another illustration to a name which has the rare honor of being so wedded to the nomenclature of disease, that it must be as immortal as the gratitude and sufferings of mankind. The essay of Dr. Bright attracted no attention to the normal function of the pancreas, until M. Bernard began his researches, when it was at once seen what a new and admirable proof of his conclusions was deducible from the information it afforded.

In the year 1846, our author placed in the stomach of a rabbit an ether solution of fat, intending to follow its changes along the intestinal track. He observed that it underwent no alteration until it had descended about twelve or fourteen inches below the pylorus, when it became emulsified, as if it had been shaken up with an alkali. At this unusual point, far below the mouth of the biliary duct, the pancreatic gland in this animal discharges its secretion. Was the change in the fat due to their reaction? A long series of experiments have answered this question. A series of experiments which are marvellous to read, if only as examples of industry, experimental enterprise, and powers of detailed observation and inductive sagacity, such as only too rarely for science are united in the grasp of any single individual.

Passing over the numberless details of his several experiments, we will endeavor to lay before the reader the general results, and the proofs on which they rest.

The pancreatic juice is obtained from animals by means of an artificial fistula. A tube inserted into the exposed pancreatic duct, directs the secretion into a gum bag, from which it is drawn as needed. The normal fluid obtained with proper precautions, is a colorless viscous fluid, without odor, and possessing a slightly saline taste, like that of blood. A constant alkalinity marks this fluid. Heat coagulates its whole bulk; nitric, sulphuric, and muriatic acids have a like effect, while alkalies do not alter it, and even redissolve the precipitates due to the acids above named. Since, in addition to these facts, we are told that acetic and lactic acids have no action upon it, we should thus have every chemical right to regard the organic material of the pancreatic juice as albumen, were it not, that unlike albumen, it is redissoluble in water after precipitation by alcohol. Slight as this chemical distinction may appear to be, the physiological resemblance between these two substances is yet more remote; another example of two organic substances, chemically allied, and widely apart as to function.

Although no fluid in the body save blood contains so large a share of albuminoid material as the pancreatic juice, every previous estimate has understated its amount. This arises from the fact, that in every such instance an abnormal secretion has been examined, in which, as M. Bernard has very well shown, the organic matters are so lessened, and the salts and water so increased, as to make it seem to approach in composition the saliva, with which it has been so long and so improperly confounded as to character and function, as to have given to its gland, the name of the abdominal salivary gland. In this peculiar organic matter our author recognizes a ferment, analogous in composition both to albumen and casein, but identical with neither, and constituting, in fact, the active and essential portion of the pancreatic juice. The functions which he supposes it to perform may be briefly stated.

The pancreatic fluid possesses the power of emulsifying oils and fluid fats, so as in some way to qualify them for admission into the blood.

The starting-point of the research out of which this proposition grew, has been already indicated. Nature had anticipated the wants of the experimenter in causing the pancreatic duct of the rabbit to enter the intestine at some distance below the bile duct. At, and after passing this point, the oils were emulsified, and the lacteal vessels began to exhibit that milky look which declares the existence of fat in their interior. A multitude of examinations of animals confirmed the general truth of this fact. If a portion of pancreatic juice be lightly shaken up with oil, at the temperature of the body, an emulsion is at once formed, so perfect in character, that it remains unchanged for an indefinite period, and is not altered by the addition of water. No other animal fluid offers a result so complete. Bile, which has some emulsifying power, separates from the oil within half an hour, and saliva, and serum, afford similar but still more imperfect results. It was naturally presumable that the highly alkaline nature of the pancreatic juice contributed to this result, since alkalies possess to a high degree the power of emulsifying and saponifying fats. As illustrative of this, we may add, that occasionally, a saliva of so alkaline a character is secreted, that it is found to produce this effect. But saliva, however alkaline, when neutralized with any acid, as for instance, gastric juice, has no power whatever to emulsify fats. On the other hand, acidulation of the pancreatic juice leaves it in full possession of this quality. It follows from this, that the alkali is not the active principle of the pancreatic fluid.

If, now, we precipitate the organic material of this secretion by alcohol, and then redissolve it in water, we shall still have a fluid capable of affecting oleaginous fluids in the manner just described,—a result which leaves us in no possible doubt as to the organic material being the effective constituent of the secretion in question. M. Bernard has also shown that this organic substance, after lengthened contact, decomposes the fats and oils into their several fatty acids and bases. Butter, for instance, is thus split into glycerine and butyric acid. This result, which is indicated by the acid reaction of the mixture upon blue litmus, appears at the close of four or five hours. So invariable are the two results, so constantly does pancreatic juice emulsify and decompose fats, and so constantly do all other gland fluids refuse to produce this change, that M. Bernard proposes it to the anatomist as a test for the actual existence of a pancreas in doubtful cases, among the lower animals. It should be understood, at the same time, that the tissue of the gland, as well as its secretion, may be made use of for this purpose. So novel an application of physiology to anatomical research is at least worthy of passing comment.

Experiments upon living animals further confirm the two propositions of M. Bernard. If the digestion of fats depends upon the pancreatic juice, any cause which deprives digestion of this fluid, will necessarily check this process, and we shall then find the unaltered fats in the small intestine, and in the alvine evacuations. Such a state of things was found to exist in the cases reported by Bright, where disease suppressed the secretion of the pancreas, and a diarrhœa of fats resulted. Although admirable as a confirmative means, the experiment thus made by disease did not fully satisfy M. Bernard, because the destruction of the gland, and the consequent loss of secretion was never quite complete. To set the question at rest, therefore, our author attempted, as Brunner had previously done, to remove the pancreas. Death inevitably followed the operation. Ligature of the ducts was next resorted to, but it was soon found that the continuity of the ducts became re-established by a curious regenerative process which M. Bernard describes in detail. This apparently ready mode of checking the flow of pancreatic secretion was, therefore, laid aside like the first one. Thus baffled by death on the one hand, and by the ingenuity of nature on the other, M. Bernard, with his usual fertility of resources, adopted a third, and eventually more successful process. It occurred to him that the secreting powers of the gland might be injured by the injection of substances into the ducts. After many failures, it was found, that when a small amount of fluid fat was thus injected, the secreting tissues of the gland became atrophied, and the ducts remained intact, like the twigs and branches of a leafless tree. In many of these cases the dogs died. In others, they became, to appearance, well, grew extremely voracious, and finally perished from marasmus. In every such instance, the animal passed the fatty portions of his food unaltered,—while, when this failed to occur, the post-mortem search always showed that the destruction of the gland had been incomplete.

Injections of blood, ether, air, and mercury, were also made use of, but they usually excited violent inflammation of the gland, and even of the peritoneum about it, so that no directly useful results were thus attained. The presence of oils, on the other hand, produced, when successfully employed, a mere atrophy of the secreting structures. From this point of view, it is impossible to think of these admirable experiments without having recalled to mind those pathological injections of gland ducts, which are sometimes the consequence of disease. Among these, the changes which the renal tubules undergo, when filled, from any cause, with coagulated fibrin, are most familiar. The loss of secreting power under these circumstances, is also well known, and we cannot avoid seeing that the solidification of the fibrin in contact with the secreting cells, may have some share in the result.

The pancreatic juice, then, is concerned in the emulsification and decomposition of fats. Disease, artificial digestions, and numerous vivisections, combine to offer us proof of this.

The fats thus prepared are now ready for absorption. Do they enter the blood through the lacteals, or by the portal vein? This is a less simple query than would at first appear, since the mode by which the fats pass through the tissues, as well as the changes undergone in this passage, are as yet among the unsolved problems of our science. It has been generally supposed that the fats, when emulsified, readily permeate the intestinal tissues to reach the vessels, or as easily pass through the cell walls of the lacteals, which are believed to possess a selective power. We are unable, however, to imitate this apparently simple physical process. When we place an emulsified oil on one side of an animal membrane and serum on the other, the aqueous portion of the emulsion goes through, unaccompanied by the fats. We know, however, that the emulsified fats of the intestine do reach the lacteals in this condition; and it has recently been shown, in Germany, that the bile assists in this, by lubricating the intestinal walls, so as to permit of the direct contact of the emulsified oils, which could scarcely occur so well were the intestine moistened with water alone.*

Whatever may be the more immediate character of the process, M. Bernard is of opinion that the bulk of the digested fats pass through the villi, and enter the lacteal vessels, in all animals belonging to the class of mammalia, while he admits that a smaller amount of oleaginous material at the same time enters the portal radicles. In birds, reptiles, and fish, the lacteal vessels have no longer a right to the name, since they do not present a milky look after the digestion of fats, and since they contain at this period no emulsified fats. In these classes of animals, the digested fats pass, by direct imbibition, into the portal system of veins. Yet, for some unknown reason, nature still seemed resolved that they should not pass wholly through the liver, and accordingly, in these animals, we find a direct route (the venous system of Jacobson) from the portal vein to the ascending vena cava—a route, by which a share of the portal blood, with its absorbed fats, is thus poured at once into the general tide of the circulation. It arises from these facts, that both in mammals, birds, reptiles, and fish, the fatty results of digestion are, for the most part, conveyed to the blood by a road different from that which is chosen for the passage of the other products of intestinal manufacture.

The anatomical facts upon which these inferences rest are, no doubt, correct; yet, while the appearance of added knowledge will always be agreeable, every thoughtful reader must see that the presence of a complex mechanism (the villi and lacteals) for fatty absorptions, in the one case, and their total absence in another, only serves to renew for us the problem of fatty absorption in a yet more complex form.

Dismissing this baffling question, M. Bernard proceeds to consider the role of the pancreas in the conversion of starch into grape sugar. Reminding us anew that this conversion is essential to absorption, and recalling the fact that the saliva fails to effect this conversion, M. Bernard shows that the pancreatic juice possesses this power over boiled starch, and that when the pancreatic secretion is absent from the canal, this part of digestion is rendered incomplete. We learn from M. Bernard's researches

* Even with the assistance of this idea, we are still at some loss to comprehend the process in question. The main difficulty, of course, lies in our inability to repeat, external to the body, an experiment apparently so simple as that of the endosmosis of emulsions. It is to be remembered, however, that other physiologists have found this more easy than M. Bernard seems to have done.

Lastly, so far as regards the passage of fats into the cells of the villi, it is to be observed, that even if we regard it as a mere physical act of imbibition, so delicate are the walls of these cells, as to put all imitative experiments at utter defiance. The conditions of the natural phenomenon are, and must be incomplete, in any attempt to reproduce it artificially.

that the saliva is chemically competent to change starch into sugar—that the gastric acid checks its action immediately, and that the pancreatic fluids complete this conversion. We shall see, further on, that they act thus upon unboiled, as well as boiled starch, provided it has been previously digested with gastric juice.

The third function of the pancreas we prefer to consider in connection with the joint action of the gastro-intestinal juices.

Preparatory to studying the conjoined action of the digestive juices, our author discusses with his usual skill, the question of the nature of the gastric acid. There seems to be no doubt, that at least in the dog, this acid is the lactic. A host of researches in Germany and elsewhere, renders the application of this fact to man rather doubtful. We may add, however, that the majority of physiologists incline to this belief, while others still believe, and with some reason, too, that chlorohydric acid is the acid of human gastric juice. Indeed, the experiments upon both sides of the case, as regards man, are to appearance, at once so decisive, and so conflicting, that we are led to suspect that lactic, butyric, and muriatic acids may all exist at times in the stomach of man. Meanwhile, it is comforting to learn, that any one of these agents is quite competent to the business of stomachal digestion. A diluted acid, and a coagulable albuminoid ferment called pepsin, are the two essentials: neutralize the acid, whatever it be, and the process stops; coagulate the pepsin, and a like result is brought about.*

This gastric juice is the first truly digestive, or solvent fluid, and M. Bernard assures us, as the result of many experiments, that the saliva does not aid it in the chemistry of digestion, however important may be its share in the mere mechanical operations essential to that process.

Our author sums up his account of the gastric juice by showing the curious analogy between its effects upon the different classes of nutriment, and those which are exerted by prolonged boiling in water. Thus boiling water melts the fats, and the stomachal temperature produces the same effect, neither agency altering their chemical composition.

The action of boiling water upon starch-cells, cracks and splits their walls, and allows the contents to become hydrated. Gastric juice brings about the same state of things, by dissolving the nitrogenous tissues which form these cell envelopes, and so freeing their contents. Usually in this case, as in that of the fats, no chemical change ensues, since the gastric juice has no sugar-making power, and the saliva has ceased to act.

* Page 414, et seq., gives an account of the variations in digestive power which are met with in specimens of gastric juice from various animals. The gastric juice of the rabbit, horse, and grain-eating bird, scarcely acts on muscle, while that of man and the dog, is alone able to dissolve the gelatinous structure of bones.

Occasionally prolonged contact with either gastric juice or boiling water converts cane sugar into grape sugar.

The effect of both of these agents upon albuminous matters is not less analogous; and, although, the result may differ in degree, it might almost be said to be identical in *kind*. In both instances the connective tissues are dissolved, so that on cooling, a gelatinous mass may be obtained in both cases, if we take the precaution to neutralize the gastric acid, which otherwise interferes with this process. The bony particles and the muscular fibres are thus disintegrated and softened, but never completely dissolved.

It is not a little amusing to be carried back in this fashion to the old view of coction, as explanatory of stomachal action.

In the condition above described the mingled results of gastric digestion enter the small intestine. Here it will be better to consider the action of the bile and pancreatic juice consecutively, although in most instances, they act together and at the same time. According to the views of M. Bernard, and most physiologists, the bile effects at once a precipitation of such gelatinous and albuminous matters as may have been previously dissolved in the stomach. At the same time the remaining albuminous matters are impregnated with bile, the fluid fats are perhaps slightly emulsified, and the amylaceous substances left unaltered. The same agency in the bile which throws down the dissolved albuminous material, also precipitates the stomachal ferment pepsin, so that reacidulation of the duodenal contents does not restore to the component gastric juice its original powers. This is very well illustrated by those cases of indigestion where bile flows into the stomach, and for the time paralyses its digestive activity. We may thus regard the act of digestion as checked by the bile. It is finally completed by the pancreatic juice, which, as we are now prepared to hear, acts upon each and all of the various nutritive and calorifacient substances upon which we feed.

If then this secretion be added to the mixture which we have supposed to exist in the duodenum, all the solid nitrogenous matters become dissolved, so that, both that which the gastric juice dissolved, and the bile threw down, and that which neither of these fluids did more than soften, attains alike a fluid condition, under the solvent influence of pancreatic juice. Every link in this chain is essential, and the pancreatic fluid has no power over the products of gastric digestion unless the bile has intervened previously, as in the rabbit, or acted conjointly with it as in man.

The changes which the pancreatic secretion effects in fats and oils do not require the presence of bile, and have been already dwelt upon sufficiently.

The conversion of starch into grape sugar requires either that the starch shall have been boiled, or that it shall have been exposed for a time to the disintegrating powers of gastric juice. Thus prepared, it undergoes this change with great readiness, and is finally converted, at least in part, into lactic acid.

In order to give continuity to our analysis of M. Bernard's opinions, we have followed the main thread in each case, often bringing together certain portions of the book which are separated in the text; but which treat of the same subject. This plan of review has obliged us to dismiss for a time several minor researches upon which our author digresses occasionally, to the temporary neglect of the principal topic of discussion.

The most novel and instructive of these lesser researches, is that in which M. Bernard establishes a test for pancreatic tissue, and juice, when in various stages of decomposition. This test consists in adding to decomposing pancreatic tissue a mixture of one part of nitric, and two of sulphuric acids, when a deep vinous color is stated to result. In an earlier stage of pancreatic decay, chlorine gas yields a similar reaction; but with the perfectly fresh gland, neither of the reagents just referred to, produces any coloration. As no other gland tissue affords any analogous reactions with these tests, the anatomist may find in them a useful ally, capable of determining the character of certain glandular tissues, whose true function in the lower animals is not always easily ascertained by an exmination of their anatomical relations and integral structures.*

Another matter of very considerable interest is the formation of artificial secretions, by digesting the gland tissues in water. In this way infusions of the various salivary glands and of the pancreas, afford fluids which in every physical and chemical character resemble the ordinary product of the glands in question. Now it can be shown that the juices of these several glands become more and more watery, and deficient in solids, the longer the flow of the natural secretion is kept up. It follows from these two sets of facts that the material from which, in each case, the secretion is formed, must have accumulated in the gland, so as to make its very tissues, so to speak, a condensed secretion, only needing solution in an alkaline water flowing from the blood in order to be eliminated in a complete and active form. In this manner the gland becomes a compact storehouse of its own secretion, and the practical effect is the same as if each gland, like the liver, were provided with a receptacle, such as the

* We have repeated with care the experiments here alluded to, and we do not think them entitled to much consideration as tests. The reaction is very variable, sometimes uncertain, and most assuredly, far less easy than the author would lead us to believe; we suspect, indeed, that there is in this part of the book some omissions, which render it far from clear. We hope hereafter to report more fully upon the subject of these tests.

gall-bladder, for the retention of its secretion until digestion demands its presence in the intestinal canal.

We can hardly pretend to have given more than an analysis of Mr. Bernard's book. Individual criticism upon a volume of experimental novelties, such as that before us, is of little value. M. Bernard's opinions are certainly at variance in many points with those of a number of eminent physiologists. He has just had the last word, and his experiments are now open to the keen criticism of repetition under the eyes of jealous rivals. On this account, therefore, we have generally left to the future and to others the task of sceptical objections. Unfortunately, our limits oblige us to dismiss without lengthened comment the chapter upon the comparative physiology of the pancreas, as well as the thoughtful and suggestive lecture upon comparative nutrition, animal and vegetable, with which the volume closes.

The literary habits of M. Dumas seem to have found partial imitators in the schools of science. A man nowadays need not write his own book. The present volume, for example, is a joint manufacture on the part of M. Bernard and two of his pupils—the professor being, of course, the thinking partner. We would be a little better pleased to have a great man's thoughts in the garments of his own proper words; we think that if our quasi author had penned the whole work fairly, we should have had more exact statements in some places, and in others, fewer of those sins of omission, which we really have not the ingratitude to point out more distinctly.

We have heard several professional friends allude disapprovingly to the unsparing resort to vivisection of which our author is said to be guilty. In this connection, M. Bernard's book suggests one or two points of interest. He remarks, on several occasions when engaged in vivisection, as to how little the animal seemed to suffer. This seeming, may be accounted for in several ways; but let us hope that the possession of a lower grade of nerve organization releases the animal from that intensity of suffering which corresponding operations would inflict upon us. There certainly appears to be no physiological warrant for believing that "the sharded beetle takes in corporeal sufferance such a pang as when a giant dies."

We are glad to observe, also, that M. Bernard occasionally makes use of chloroform when about to dissect the living animal. It might have been used more often, since in most cases its employment is, in every point of view, an advantage. Certainly, it is not unpleasant to think that science, which invented these tortures, has at last come to their relief; so that now we may feel the more freedom in sacrificing painlessly, and for useful ends, the life of the beast, over whom nature has given us a dictatorship so imperial.

Occasionally, some physiologist finds fault with vivisection as a means of research, not for moral reasons, but because it is so open to fallacies. Unluckily, this objection is applicable to every method of research, so that the better the means, as a whole, the greater becomes the chance of fallacious observation or interpretation. In the telescope, a higher magnifying power and better lenses at once increase the possible sources of error; yet, on this plea, no one would feel inclined to lay aside an improved means, whose full use was to be bought at the cost of a little more care and labor. The real want is in the observer, and not in the means of observation. The genius of experimentally guided induction is the rarest gift of nature, and with vivisection the difficulties multiply beyond the control of most men, since here we reason upon facts which we have actually assisted to create. The results of vivisection, as of other means of research, may be turned but too easily into the ready channels of our own preconceptions, so that docility in following the hints offered us through experiment is here, as elsewhere, the real want. Yet the eyes of truth, like those of a fair portrait, follow us always, could we but learn to look her steadfastly in the face.

But the subject has tempted us far enough. We cannot better take leave of it than with the indorsement of the Father of English physiology. When, says William Harvey, I first gave my mind to vivisection as a means of discovering the uses and motions of the heart, I found the task so truly arduous, so full of difficulties, that I was almost tempted to think, with Fracastarius, that the motion of the heart was only to be comprehended by God. At length, and by using greater and daily diligence, having frequent recourse to vivisections, employing a variety of animals for the purpose, and collating numerous observations, I thought that I had attained to the truth.

From his time to ours, nature has paid out her secrets miserly, in the hard coin of anguish.*

* The life of J. Reid, of Edinburgh, contains an excellent summary of the arguments for and against the practice of vivisection. This sagacious physiologist, well known for his researches upon the nerves of the tongue and throat, died of a cancer of the very parts whose functions he had illustrated by years of patient labor. Rendered morbid by disease and weakness, he spoke of his malady as a visitation sent to punish him for the free use which he had made of vivisection, as a means of research. Certainly there is no more painful story than his in all the annals of medical biography.

